# 439. Anal Dysplasia Screening Cascade within a Single-Center Outpatient HIV Clinic

**DOI:** 10.1093/ofid/ofac492.514

**Published:** 2022-12-15

**Authors:** Chelsea Modlin, Danica Rockney, Jerald Cherian, Diana Zhong, Sonya Krishnan, Styliani Karanika, Joyce Jones

**Affiliations:** Johns Hopkins University School of Medicine, Baltimore, Maryland; Johns Hopkins University, Baltimore, Maryland; Johns Hopkins University School of Medicine, Baltimore, Maryland; Johns Hopkins University School of Medicine, Baltimore, Maryland; Johns Hopkins University School of Medicine, Baltimore, Maryland; Johns Hopkins University School of Medicine, Baltimore, Maryland; Johns Hopkins University School of Medicine, Baltimore, Maryland

## Abstract

**Background:**

HIV-positive men who have sex with men (MSM) are recommended to have anal Pap tests annually to screen for high-grade anal squamous intraepithelial lesions (HSIL). In this population, removal of HSIL reduces the risk of progression to anal cancer. This study aims to assess the effectiveness of the anal dysplasia screening cascade in a single-center outpatient clinic.

**Methods:**

We conducted a single-center retrospective cohort study at Johns Hopkins. All adult HIV-positive MSM patients who had in-person encounters in the Bartlett Clinic between October 1, 2019-April 30, 2021 were included. Clinical information and dates of anal pap smears and HRAs were provided via an Epic data extraction. Manual chart review was conducted for anal pap and HRA pathology results. Descriptive analysis was performed. The primary outcome was successful navigation of the institutional anal dysplasia screening cascade, defined as annual anal Pap testing and completion of HRA if pap results were abnormal. This study was IRB exempt.

**Results:**

779 HIV-positive MSM patients were seen for in-person visits during the study period. 24% (190/779) of patients underwent at least one screening anal Pap, with 29 having multiple anal pap smears leading to a total of 219 anal pap smears done. 40% (87/219) were abnormal. Among those with an abnormal pap, 72% (63/87) had a referral for HRA placed but only 26% (23/87) underwent HRA. 39% (9/23) of HRAs identified HSIL in patients with lower-grade lesions on Pap test and one patient with HSIL on Pap had anal cancer detected on HRA. In total, 120 patients navigated the anal cancer screening cascade appropriately. This compromised 15% (120/779) of eligible MSM patients seen for at least one in-person clinic visit and 63% (120/190) of patients who underwent an anal Pap.

**Conclusion:**

Completion of a screening cascade for anal dysplasia among HIV-positive MSM at our clinic was low. The largest gaps were seen in initiating annual anal Pap screening and scheduling referrals for HRA in surgical subspecialty clinics. Increased attention and clinic resources should be directed toward narrowing these gaps to ensure HSIL and anal cancers are detected early within this high-risk population.

**Disclosures:**

**All Authors**: No reported disclosures.

